# Safety and efficacy of prophylactic anticoagulation versus therapeutic anticoagulation in hospital‐admitted COVID‐19 patients: A systematic review and meta‐analysis of randomized controlled trials

**DOI:** 10.1111/crj.13568

**Published:** 2022-12-26

**Authors:** Robin Rauniyar, Sandip Kuikel, Aman Mishra, Rohit Rauniyar, Shikha Yadav, Sahil Thapaliya, Amit Sharma Nepal, Rahul Rauniyar

**Affiliations:** ^1^ Maharajgunj Medical Campus Tribhuvan University Institute of Medicine Kathmandu Nepal; ^2^ Internal Medicine McLaren Flint/Michigan State University (MSU) Flint Michigan USA; ^3^ Nepalgunj Medical College Kathmandu University Kathmandu Nepal; ^4^ Internal Medicine, The Wright Center for Graduate Medical Education Scranton Pennsylvania USA

**Keywords:** anticoagulation in COVID‐19, COVID‐19, prophylactic anticoagulation, SARS‐CoV‐2, therapeutic anticoagulation

## Abstract

**Background:**

COVID‐19 disease‐related coagulopathy and thromboembolic complication, an important aspect of the disease pathophysiology, are frequent and associated with poor outcomes, particularly significant in hospitalized patients. Undoubtedly, anticoagulation forms a cornerstone for the management of hospitalized COVID‐19 patients, but the appropriate dosing has been inconclusive and a subject of research. We aim to review existing literature and compare safety and efficacy outcomes of prophylactic and therapeutic dose anticoagulation in such patients.

**Methods:**

We did a systematic review and meta‐analysis to compare the efficacy and safety of prophylactic dose anticoagulation when compared with therapeutic dosing in hospitalized COVID‐19 patients. We searched PubMed, Google Scholar, EMBASE and COCHRANE databases from 2019 to 2021, without any restriction by language. We screened records, extracted data and assessed the risk of bias in the studies. RCTs that directly compare therapeutic and prophylactic anticoagulants dosing and are not placebo‐controlled trials were included. Analyses of data were conducted using the Mantel–Haenszel random‐effects model (DerSimonian–Laird analysis). The study is registered with PROSPERO (CRD42021265948).

**Results:**

We included three studies in the final quantitative analysis. The incidence of thromboembolic events in therapeutic anticoagulation was lower in comparison with prophylactic anticoagulation in hospitalized COVID‐19 patients and reached statistical significance [RR 1·45, 95% CI (1.07, 1.97) *I* ^2^ –0%], whereas major bleeding as an adverse event was found lower in prophylactic anticoagulation in comparison with therapeutic anticoagulation that was statistically significant [RR 0·42, 95% CI(0.19, 0.93) *I* ^2^ –0%].

**Conclusion:**

Our study shows that therapeutic dose anticoagulation is more effective in preventing thromboembolic events than prophylactic dose but significantly increases the risk of major bleeding as an adverse event. So, the risk–benefit ratio must be considered while using either of them.

## Graphical abstract

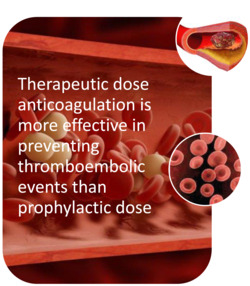

## 1 INTRODUCTION

Coronavirus disease 2019 (COVID‐19), first reported in Wuhan, China, in December 2019, evolved as a pandemic and has been a major cause of morbidity and mortality globally.^1^ COVID‐19, caused by severe acute respiratory syndrome coronavirus 2 (SARS‐CoV‐2) constitutes a manifold expression affecting the respiratory system largely along with gastrointestinal, haematological, otorhinolaryngological and neurological involvement.^2^, ^3^

Previous observational studies have shown that infection with SARS‐CoV‐2 is associated with hypercoagulability.^4^, ^5^, ^6^, ^7^ Prothrombin prolongation, low antithrombin activity and increased fibrinogen and d‐dimer levels are the coagulation abnormalities associated with COVID‐19 infection. However, the exact mechanisms of these abnormalities in coagulation and fibrinolysis in patients with COVID‐19 are unknown.^8^, ^9^ These abnormalities result in both arterial and venous thrombotic events leading to a higher risk of morbidity and mortality.^10^ The diffuse lung injury as a result of microvascular thrombosis eventually leads to acute respiratory failure in patients with COVID‐19.^11^

Though studies are ongoing, there is limited availability of data for use of anticoagulation in patients with COVID‐19. Many observational studies have shown the rationale for both therapeutic and prophylactic doses of anticoagulation with low molecular weight heparin (LMWH) to reduce morbidity and mortality in these patients.^12^, ^13^, ^14^ However, the efficacy and safety of both doses are still unknown.

In this systematic review and meta‐analysis, we aim to summarize the safety and efficacy of prophylactic anticoagulation against therapeutic anticoagulation in patients hospitalized with COVID‐19. Some randomized controlled trials (RCTs) are performed comparing the safety and efficacy of either in patients with COVID‐19 infection, but there was an urgent need for systematic review and meta‐analysis to make the conclusions more robust.

## 2 METHODS

### 2.1 Search strategy

This study was done in accordance with the Preferred Reporting Items for Systematic Reviews and Meta‐Analyses (PRISMA) statement.

We performed a systematic search of RCTs comparing the outcome of prophylactic and therapeutic anticoagulants in hospitalized COVID‐19 patients. PubMed, EMBASE and COCHRANE were the online databases used to search for studies. We utilized Google Scholar and reference of other manuscripts to search for additional studies. We developed a search strategy and adjusted it for each engine using different keywords: prophylactic anticoagulation, therapeutic anticoagulation AND (COVID‐19 OR coronavirus OR coronavirus disease OR coronavirus disease‐19 OR severe acute respiratory syndrome OR SARS‐CoV‐2). The exact search strategy used in one of the databases (PubMed) is provided as a supplementary file. The references of the included studies were reviewed to search for additional studies. Databases were searched on 7 July 2020, and there were no language restrictions applied during the search, screen or the selection process of studies. The included studies were published between 2019 and 2021. This systematic review and protocol were registered online in PROSPERO (CRD42021265948).

### 2.2 Study selection

We developed inclusion and exclusion criteria for the studies as follows:

- Inclusion criteria
  - Randomized controlled clinical trials
  - Comparing therapeutic versus prophylactic anticoagulation in COVID‐19 patients
  - Patients may be critically or non‐critically ill
- Exclusion criteria
  - Other study types except for RCTs

Based on the aforementioned criteria, title and abstract screening were done by two independent reviewers [Robin Rauniyar (RR1) and AM] using Covidence. The third reviewer (SK) reviewed all the studies of conflict. The full‐text review of studies qualifying inclusion criteria after the title and abstract screening was done by another reviewer [Rohit Rauniyar (RR2)].

### 2.3 Data extraction and quality assessment

Two authors [SY and Rahul Rauniyar (RR3)] independently extracted the data in MS Excel version 2016. An Excel sheet template was made, and data were extracted under the following heading: author name, study year, number of participants and study design. Under each study, treatment and control arms were made, and safety and efficacy outcomes were extracted and recorded. Safety outcome of concern included the occurrence of major bleeds, and the efficacy outcome was the occurrence of thromboembolism (thromboembolism defined as occurrence of thrombosis in any vessel, artery or vein, with or without embolism). The extracted data were again reviewed by another reviewer (ST).

The qualities of RCTs were assessed by two authors (RR1 and ASN) using the Cochrane Risk of Bias Tool.^15^ The risk of bias has been assessed across six domains: (1) random sequence generation, (2) allocation concealment, (3) blinding of participants and personnel, (4) blinding of outcome assessment, (5) incomplete outcome data and (6) selective reporting.

### 2.4 Data analysis

Pooled proportions, risk ratio (RR) and 95% confidence intervals of safety and efficacy outcomes were generated from included RCTs. Forest plots of comparative RRs (prophylactic vs. therapeutic) were created using the RevMan 5·4 software. Analyses were conducted using the Mantel–Haenszel random‐effects model (DerSimonian–Laird analysis).^16^ Heterogeneity between trials was assessed by visual inspection of forest plots and by the percentage of total variation across studies above chance alone (*I* ^2^ statistic).^17^

## 3 RESULTS

### 3.1 Study selection and characteristics

**FIGURE 1:**
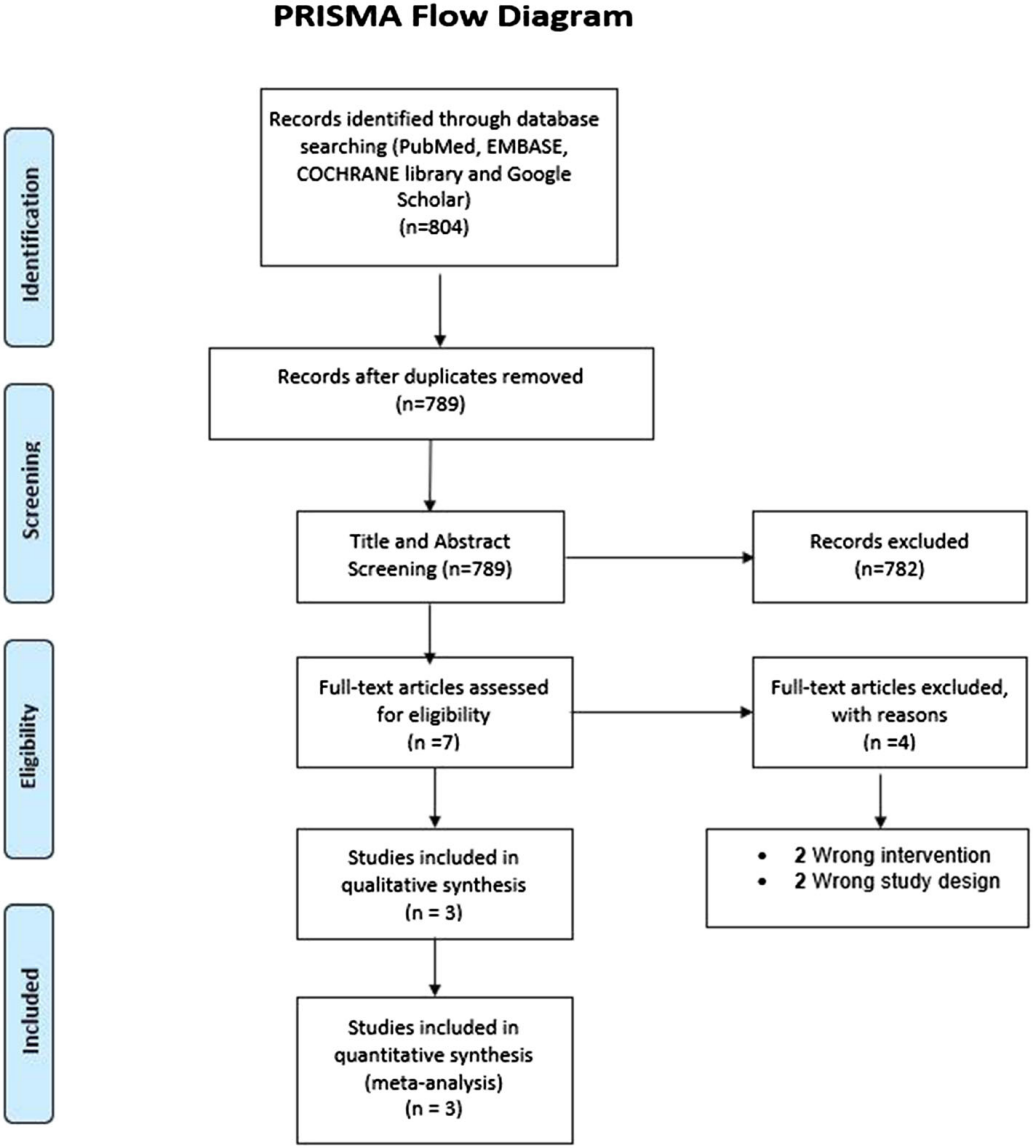
PRISMA flow diagram showing the study selection process

A total of 804 studies were identified on initial search on different databases, of which 15 duplicates were removed. After title and abstract screening of 789 studies, seven studies were eligible for full‐text review. Finally, a total of three RCTs were included in our systematic review and meta‐analysis. The study review process is depicted in the PRISMA flow diagram (Figure 1).

The total number of participant patients in the RCTs (*n* = 3) included in our study was 1732.^18^, ^19^, ^20^ The RCTs randomized the patients with COVID‐19 into two groups. One of the groups was treated with a prophylactic dose of anticoagulants, whereas the other group was treated with a therapeutic dose of anticoagulants. The total number of patients receiving prophylactic dose anticoagulants is 878, and the total number of patients receiving therapeutic dose anticoagulants is 854 (Table 1).

**TABLE 1:** Baseline characteristics of the included studies

Study	Year	No. of patients	Intervention	No. of patients	Age	Male sex %	Bleeding	Thrombosis
Lawler et al	2021	1098	Prophylactic	564	61.7 ± 12.5	67.9	13	62
			Therapeutic	534	60.4 ± 13.1	72.2	20	38
Lopes et al	2021	614	Prophylactic	304	56.7	90	7	30
			Therapeutic	310	56.5	70	26	23
Lemos et al	2020	20	Prophylactic	10	58 ± 16	62	0	2
			Therapeutic	10	55 ± 10	58	0	2

### 3.2 Thromboembolism

**FIGURE 2:**
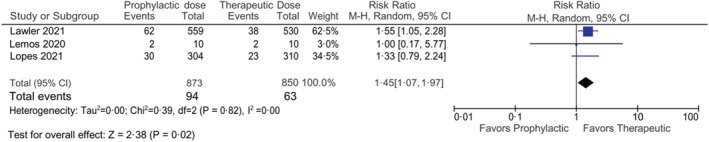
Forest plot showing the comparison of thromboembolic events in prophylactic vs therapeutic anticoagulant dose

Pooled data from the included RCTs showed the occurrence of thromboembolism in 94 out of 873 patients in the prophylactic anticoagulation group and 63 out of 850 patients in the therapeutic anticoagulation group. The pooled results showed that the occurrence of thromboembolism in therapeutic anticoagulation was lower in comparison with prophylactic anticoagulation in COVID‐19 patients that reached statistical significance [RR 1.45, 95% CI (1.07, 1.97) *I* ^2^ –0%] (Figure 2).

### 3.3 Major bleeding

**FIGURE 3:**
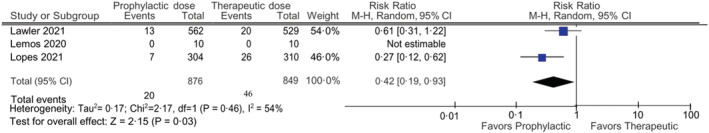
Forest plot showing the comparison of bleeding events in prophylactic versus therapeutic anticoagulant dose

Pooled data from the included RCTs showed the occurrence of bleeding in 20 out of 876 patients in the prophylactic anticoagulation group and 46 out of 849 patients in the therapeutic anticoagulation group. The pooled results showed that bleeding as an adverse event was lower in prophylactic anticoagulation in comparison with therapeutic anticoagulation that reached statistical significance [RR 0.42, 95% CI (0.19, 0.93) *I* ^2^ –54%] (Figure 3).

### 3.4 Quality assessment

**FIGURE 4:**
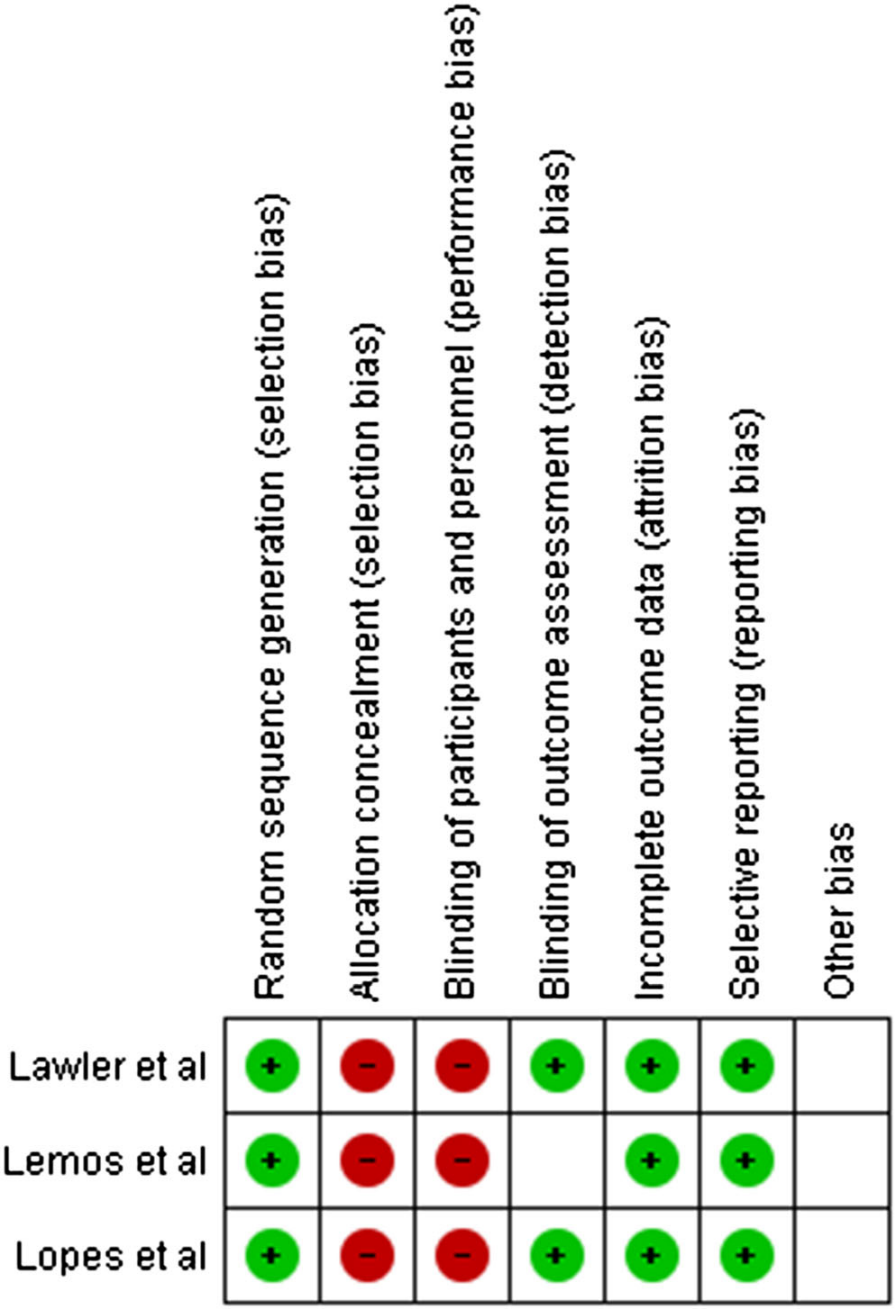
Quality assessment of the included studies

Results from the quality assessment are provided in the table below. All the studies included did not report on allocation concealment and blinding of participants and personnel. All studies used random sequence generation and reported on blinding of outcome assessment. None of the studies reported incomplete outcome data, and no evidence of selective reporting was found in any studies (Figure 4).

## 4 DISCUSSION

The consensus guideline suggests the usage of parenteral LMWH for thromboprophylaxis in COVID‐19.^21^ However, there are no consensus data regarding doses and safety and efficacy of different doses of LMWH. RCTs have been conducted to compare the efficacy of prophylactic versus therapeutic doses of LMWH in patients with COVID‐19, and this study summarizes the findings of such RCTs. To the best of our knowledge, this is the first systematic review and meta‐analysis of RCTs comparing the efficacy and safety outcome of prophylactic dose directly compared with therapeutic dose anticoagulation in COVID‐19 patients.

The prevalence of arterial or venous thromboembolism (VTE) in hospitalized COVID‐19 patients varies widely, ranging from 15.2% to 69% among different studies.^22^, ^23^ A recent meta‐analysis of such studies showed the pooled prevalence of VTE in such patients receiving therapeutic or prophylactic dose anticoagulation to be 31%.^24^ Despite the various studies reporting varied prevalence rates of VTE in COVID‐19, the exact mechanism of its occurrence has not been described. However, many theories have been postulated regarding its pathogenesis. A possible mechanism postulated by various studies is cytokine storm due to viral infection. This theory is based on the observation of high plasma levels of cytokine in patients with COVID‐19 patients compared with healthy individuals.^25^, ^26^, ^27^ Another plausible explanation of the hypercoagulability in patients with COVID‐19 is the presence of high levels of interleukin‐6 (IL‐6) and supported by the potential benefit of IL‐6 inhibitors like tocilizumab in severe COVID‐19 patients.^26^, ^28^

There have been many proposed treatments for the COVID‐19 patients, of which remdesivir, hydroxychloroquine, steroids and anticoagulants have been studied the most. So far only dexamethasone has been shown to have 28‐day mortality benefit in COVID‐19 patients in the RECOVERY trial.^29^ Tang et al found no difference in 28‐day mortality for the general population of anticoagulant users and non‐users overall, but patients with sepsis‐induced coagulopathy score ≥4 and those patients on heparin for ≥7 days had lower mortality than those not on anticoagulants (40.0% vs. 64.2%, *p* = 0.029).^14^

Our meta‐analysis shows that the therapeutic dose anticoagulation has better efficacy that was statistically significant in terms of prevention of occurrence of thrombosis when compared with prophylactic dose. Similarly, our safety outcome measured in terms of major bleeding episodes suggests that prophylactic dosing has a lower incidence of major bleeding as an adverse event that reached statistical significance when compared directly with therapeutic dosing. The major bleeding described in the RCTs is per the definition of the International Society on Thrombosis in which major bleeding in non‐surgical patients is defined as having (1)fatal bleeding, and/or (2)symptomatic bleeding in a critical area or organ, such as intracranial, intraspinal, intraocular, retroperitoneal, intra‐articular or pericardial or intramuscular with compartment syndrome, and/or (3) bleeding causing a fall in haemoglobin level of 20 g/L (1.24 mmol/L) or more, or leading to transfusion of two or more units of whole blood or red cells. These findings are consistent with a recent systematic review that aimed to explore the association between therapeutic dose anticoagulation and its effect on mortality in COVID‐19 patients. It summarized the findings of nine studies, among which three retrospective cohort studies reported a reduction in the mortality rate, while six other studies showed no mortality benefits among COVID‐19 patients treated with therapeutic dose of anticoagulation.^30^ In contrast to the findings from the RCTs included in our study, our findings showed a significant difference in efficacy between the prophylactic and therapeutic doses. The study by Lemos et al reported that intubated patients on therapeutic dose of anticoagulants were on mechanical ventilation for fewer days, but the small number of patients enrolled (*n* = 20) in the trial hampers the confidence of its results.^18^ Similar to the findings of our meta‐analysis, all three studies reported a decreased incidence of bleeding with prophylactic dosing.^19^, ^20^

Although this review and meta‐analysis tries to solve the clinical question of appropriate dosing of anticoagulation in COVID‐19 patients, there is still a need for other RCTs directly comparing the two arms with appropriate outcomes. This study supports the use of therapeutic dose anticoagulation over prophylactic dose due to its significant decrease in the occurrence of thrombosis as an efficacy outcome, but there is a significantly increased incidence of major bleeding as an adverse event with therapeutic dose of anticoagulants. So, there must be a risk–benefit analysis before using either of the doses considering the comorbid conditions, which might increase the propensity of bleeding.

The major strength of this systematic review and meta‐analysis is that only RCTs have been included in the quantitative analysis, so the strength of association could be accurately measured. Similarly, the included RCTs directly compare therapeutic and prophylactic dosing and are not placebo‐controlled trials that give better strength of evidence. The major limitation of our study is the number of RCTs included, and heterogeneity in the number of participant patients in the included trials as one of the RCTs included a small number of patients (*n* = 20).^18^ The heterogenicity of the results on bleeding was high with *I* ^2^ 54%; we used random‐effects model to analyse the pooled result, but leave‐one‐out study analysis could not be performed due to limited number of studies included in the analysis. Also, the exact dose of anticoagulant used as prophylactic and therapeutic dose in COVID‐19 patients have not been mentioned in all the RCTs included in our review.

## CONFLICT OF INTEREST

The authors declare no competing interests.

## AUTHOR CONTRIBUTIONS

Robin Rauniyar (RR1), Sandip Kuikel (SK), Aman Mishra (AM), Rohit Rauniyar (RR2) and Rahul Rauniyar (RR3) designed the study. RR1 and Shikha Yadav(SY) designed and ran the literature search. Sahil Thapaliya (ST) and AM performed the title and abstract screening. The conflicts were resolved by Amit Sharma Nepal(ASN). The full‐text review was done by RR2. RR1 and RR3 extracted the data and analysed them. RR3 assessed the risk of bias. RR1, SK and AM wrote the manuscript. All authors provided critical conceptual input, analysed and interpreted data and critically revised the report.

## Data Availability

The available data of the study will be shared on reasonable request to the corresponding author (RR1) via email after the publication of the study.
